# P-1343. Prevalence and Clinical Outcomes of Methicillin-resistant Coagulase Negative Staphylococci (MRCONS) Infection in Northeast Region of Thailand

**DOI:** 10.1093/ofid/ofaf695.1531

**Published:** 2026-01-11

**Authors:** Pongpisit Wongjirattikarn, Atibordee Meesing

**Affiliations:** srinagarind hospital, bangkok, Krung Thep, Thailand; Khon Kaen university, A. Muang, Khon Kaen, Thailand

## Abstract

**Background:**

Methicillin-resistant coagulase-negative *staphylococci* (MRCONS) are increasingly recognized for their impact on healthcare, but data on their prevalence and outcomes in Thailand remain limited. These pathogens are often colonizers rather than true infection agents, and information on drug resistance and associated mortality is scarce.

Objectives To assess the prevalence of MRCONS infections and their clinical outcomes, including 28-day mortality, hospital and ICU lengths of stay, and vancomycin minimum inhibitory concentrations (MICs).

**Figure:**
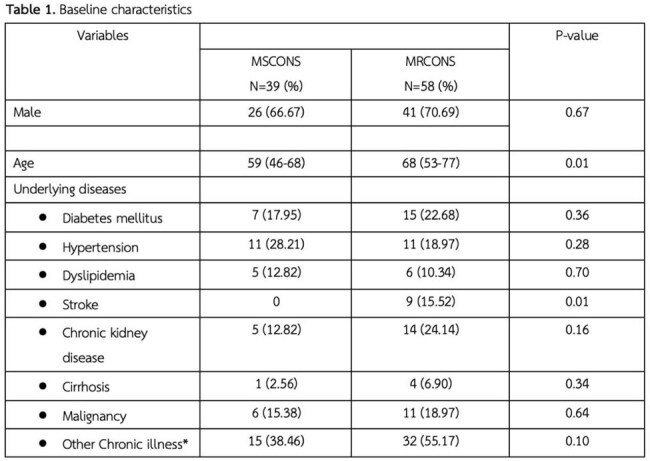
*Other chronic illness: Systemic autoimmune disease: 4 (8.51%), Heart disease: 15 (31.91%), Gastrointestinal disease: 1 (2.12%), Benign prostatic hyperplasia: 1 (2.12%), HIV infection: 1 (2.12%), Bone and joint disease: 13 (27.65%), Hematologic disease: 4 (8.51%), Neurological disease: 6 (12.76%), Tuberculosis: 1 (2.12%), Chronic viral hepatitis B infection: 1 (2.12%)

**Figure:**
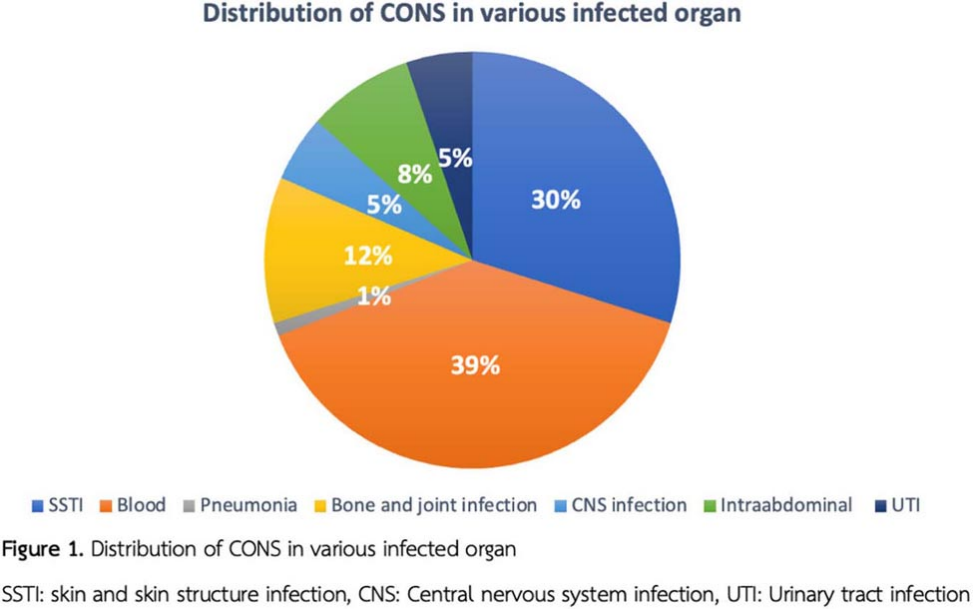
SSTI: skin and skin structure infection, CNS: Central nervous system infection, UTI: Urinary tract infection

**Methods:**

This retrospective study (January 2018–October 2023) included patients aged over 18 with confirmed CONS infections based on culture results and clinical signs.

**Figure:**
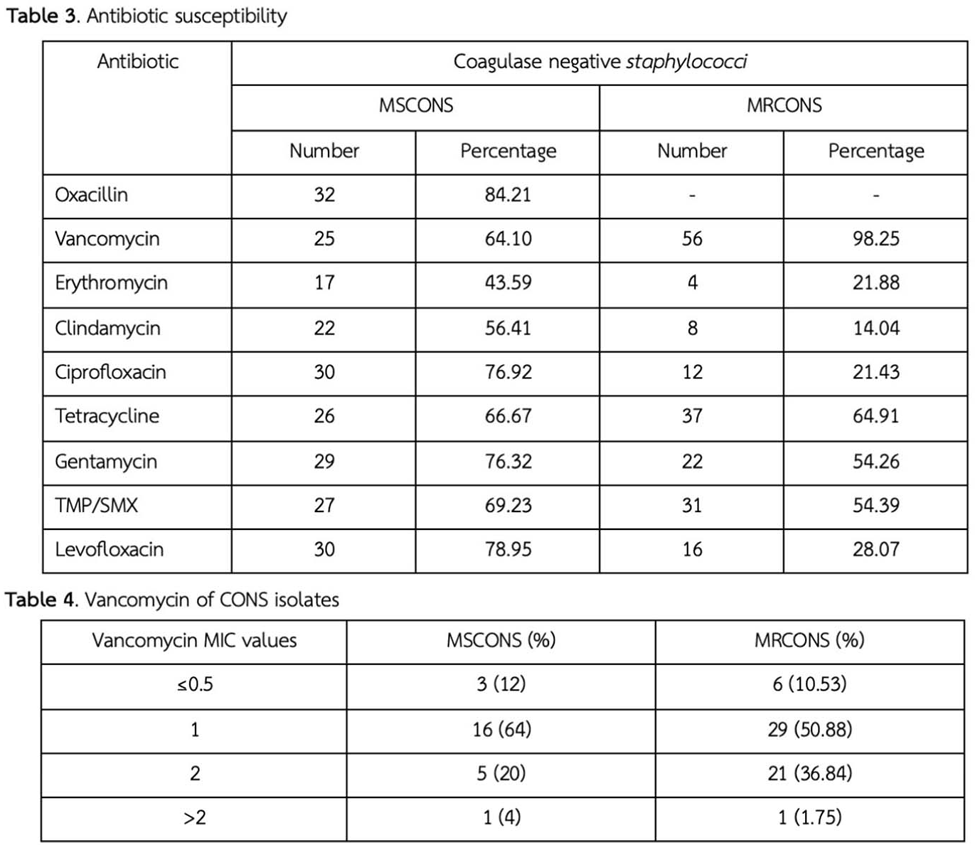

**Figure:**
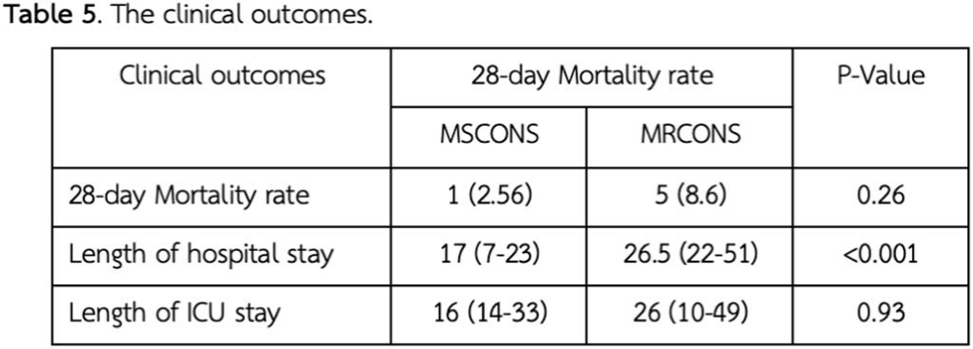

**Results:**

Among 97 patients with true CONS infections (median age: 59 [IQR 46-68] years for Methicillin-susceptible coagulase-negative *staphylococci* (MSCONS), 68 [IQR 53-77] years for MRCONS), 59.75% had MRCONS infection. Underlying conditions were more frequent in the MRCONS group, particularly chronic kidney disease (25.45% vs. 13.51%). The 28-day mortality rate was higher in the MRCONS group (9.8%) compared to the MSCONS group (2%), though the difference was not statistically significant (P = 0.26). Hospital stays were significantly longer for MRCONS patients, with a median duration of 26.5 days (IQR 22–51) compared to 17 days (IQR 7–23) in the MSCONS group (P < 0.001). ICU stays followed a similar pattern, with median durations of 26 days (IQR 10–49) for MRCONS group and 16 days (IQR 14–33) for MSCONS group (P = 0.93). The vancomycin MICs of the MRCONS group were categorized as follows: < 0.5, 1, 2, and > 2, with corresponding frequencies of 10.53%, 50.88%, 36.84%, and 1.75%, respectively.

**Conclusion:**

MRCONS infections accounted for over half of CONS cases, significantly prolonging hospital stays and showing a trend toward higher mortality and ICU stays. Physicians should remain alert to resistant strains in CONS infections.

**Disclosures:**

All Authors: No reported disclosures

